# Morphological Changes of Parotid Gland in Experimental Hyperlipidemia

**DOI:** 10.1155/2011/928386

**Published:** 2011-03-21

**Authors:** Ioanna D. Daskala, Christina C. Tesseromatis

**Affiliations:** ^1^Faculty of Dentistry, University of Athens, 11527 Goudi, Greece; ^2^Medicine in Pharmacology Department, Faculty of Medicine, University of Athens, 11527 Goudi, Greece

## Abstract

*Objective*. The aim of this study was to investigate the role of hyperlipidemia in the microstructure of parotid gland and its possible amelioration through statin treatment on Wistar rats. *Methods*. Forty Wistar rats (111.06 ± 3.36 g) were divided into 4 groups (A1, A2 controls, B1, B2 experimental). Groups A1 and A2 consumed normal cereal rodents diet during the experimental hyperlipidemic mixture. The A2 and B2 were treated with simvastatin (Zocor) 40 mg/kg/daily p.o. for 3 months. *Results*. Total cholesterol, triglycerides, high density lipoproteins, and low density lipoproteins were increased in groups B1 and B2 while the parotid weight was decreased. The histological findings demonstrated changes in the parotid gland morphology of the B1 and B2, such as the presence of chronic inflammation, fibrosis, lipocytes, and foci of lymphocytic infiltration. *Conclusions*. The influence of statin tended to predominate over the chronic inflammation while the lipocytes were decreased and remodelling of the parotid's structure occurred.

## 1. Introduction

Hyperlipidemia is characterized by elevated levels of plasma total cholesterol (TC), triglycerides (TG), and low-density lipoprotein cholesterol (LDLC) [1, 2]. Patients with hyperlipidemia are considered to be at high risk to develop lipids disorders that are associated with immune system deficiency, promoting manifestations including fatty degeneration of parotid parenchyma and parotid duct enlargement [3–6]. Various diseases could be associated with parotid gland swelling, such as primary or secondary Sjogren Syndrome (SS), diabetes mellitus, Wegener's granulomatosis, sialadenitis, and infections [7–9].

Recent studies have revealed that patients with SS and treated for hyperlipidemia may present a resolution of parotid symptoms [10]. Few clinical studies demonstrated that children and adolescents with hypercholesterolemia present endothelial disfunction [11, 12]. Simvastatin reducing TC and LDL-C concentrations restores endothelial function during a short period of time [11, 12]. Statin benefits are derived from reductions in atherogenic lipoprotein levels and increases in high-density lipoprotein cholesterol (HDL-C), [13, 14].

Abnormal fat deposition in the major salivary glands such as parotid gland could be detected by using short-inversion-time inversion recovery (STIR) and fat-saturation MR sequences and CT values. All three in vivo techniques confirm premature deposition of fat in the major salivary glands while the severity of fat deposition could be correlated with impaired rates of salivary flow in these patients [15–17]. MR imaging findings of salivary glands in patients with hyperlipidemia include enlargement of parotid gland, lipid infiltration, and impaired salivary flow, whereas sialography of parotid gland revealed normal findings [15–17]. Monitoring of fat deposition might be a very useful weapon in diagnosis any parotid gland's disease [15–18].

The aim of this study was to investigate the role of hyperlipidemia in the parotid gland's microstructure of Wistar rats and the influence of simvastatin treatment.

## 2. Materials and Methods

Forty female, specific–pathogen-free Wistar rats aged 8–10 weeks (mean body weight 111.06 ± 3.36 g) were used and divided into the following four groups:

A1 control: normal cereal rodents diet (*n* = 10),
A2 control: normal cereal rodents diet (*n* = 10),
B1 experimental: special diet (*n* = 10),
B2 experimental: special diet (*n* = 10).

Groups B1 and B2 were treated with high fat diet in order to investigate its influence in rats (group B1). Normal cereal rodents' diet contained 100% cereals while hyperlipidemic diet consisted of 50% cereals, 20% butter, 10.5% sucrose, 10% casein, 2.5% cholesterol, 1% vitamins, 0.15% propylthiouracil and 0.1% choline [19]. Moreover, groups A2 and B2 received simvastatin (Zocor was gratefully offered by Vianex) 40 mg/kg/daily peros for 3 months via gastroesophageal cathether.

The animal experiment was performed in accordance with the institutional and national guidelines for the care and the use of experimental animals and, therefore, in compliance with the European Communities Council Directive of 24 November 1986 [20]. All animals were sacrificed under general anaesthesia and blood samples from carotid vessel were collected in order to examine the serum lipids such as TC, HDL, LDL, and TG. Parotid gland was isolated, removed, and examined. The purpose was to notify any difference in the organs' macro and microstructure. The parotid glands were preserved in 10% buffered formaldehyde fixative solution (Merck) for further histological examination. Sections of parotids' specimens were stained with haematoxylin-oesin, and a full histological investigation was carried out.

Furthermore, the morphometrics features were evaluated using a microscope Bx42 Olympus, a camera Altra 20 Olympus, and image's analysis with Image —Pro Plus. Moreover, based on the morphometric findings, a statistical analysis was performed using Excel-Student *t*-test. Biochemical features were statistically analyzed by performing SPSS Kruskal-Wallis Test and Mann-Whitney Test.

## 3. Results

### 3.1. Laboratory Findings

The hyperlipidemic diet in group B1 leads to a statistically significant increase of TC, HDL, HDL, and TG (*P* < .001) and to an insignificant increase of parotid gland weight compared to group A1 (normal cereal diet) and A2 (normal cereal diet + simvastatin), (Table 1).

 Furthermore, the simvastatin treatment in group B2 (hyperlipidemic diet + simvastatin) compared to group B1 decreased the serum levels of TC, HDL, LDL, TG and parotid gland in a statistically significant way (*P* < .001), (Table 1). 

No changes were observed in group A2 compared to group A1 concerning the serum lipids profile and the parotid gland weight (Table 1). Group A2 presented a statistically insignificant decrease of parotid gland weight compared to group B1 and group B2 (Table 1). Group B2 in comparison to group A2 the serum levels of TC, HDL, LDL, TG, and liver weight were increased in a statistically significant way (*P* < .001), (Table 1). 

Finally, as far as the atheromatic index in group B1 and group B2 was concered, it should be underlined that there was a statistically insignificant difference (Table 1).

### 3.2. Histological Findings via Morphometry

According to histological findings, different state of lipid infiltration of the parotid gland specimens, stained with haematoxylin-oesin, in hyperlipidemic animals in group B1, B2 was observed. In addition, chronic inflammation, mild fibrosis, lypocytes, foci of lymphocytic infiltration nuclei abnormalities and diversity of vessel wall thickness were recorded [4, 18].

**Figure 1:**
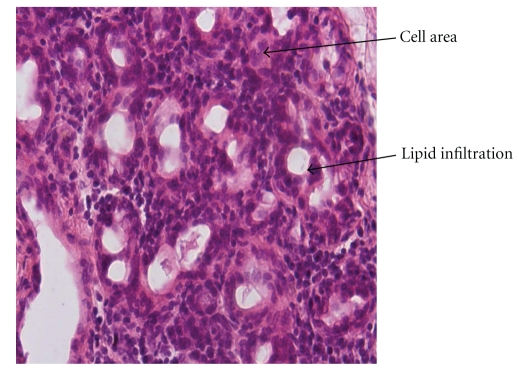
In group B1, lipid infiltration of the parotid parechyma, presence of lipocytes and nuclei abnormalities (increased cell area and duct's diameter, decreased duct's number) were observed (haematoxylin eosin— original magnification ×400).

**Figure 2:**
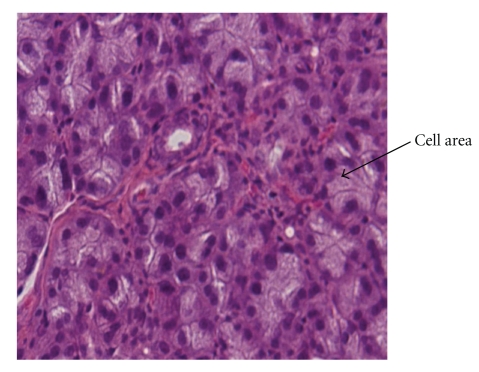
In group B2, there were not significant alterations in the cell area or the number and the diameter of the ducts of the parotid gland (haematoxylin eosin—original magnification ×400).

Moreover, based on the morphometrical analysis of the histological features, our research resulted in the following discoveries:

- Animals of group B1 compared to animals of group A1, demonstrated a statistically significant increase of cell area and duct's diameter while the duct's number was decreased (*P* < .001, Figure 1, Table 2).
- Animals of group B2 compared to animals of group A1, revealed statistically insignificant alterations in the cell area, the number and diameter of the ducts (*P* > .01, Figure 2, Table 2).

### 3.3. Discussion

The goal of this research was to evaluate the microstructural alterations of parotid gland in experimental hyperlipidemia and the possible amelioration through statin treatment in Wistar rats. According to the biochemical data hyperlipidemic diet leads to an elevated serum lipid profile that might be responsible for parotid gland swelling as it is referred from Goldman and Julian, Kaltreider and Talal, Manganelli et al., and Tretbar [3, 4, 6, 19]. In addition, Izumi et al. suggest that parotid gland is more affected by hyperlipidemia than submandibular gland [16, 17]. The above results are in accordance with the findings of Sheikh et al., suggesting a close relationship between parotid gland swelling and high levels of triglycerides in plasma [10, 16, 17].

Moreover, it is not well known how hyperlipidemia causes parotid gland swelling. Larsson et al. used the Folch method in order to extract the lipids from parotid, submandibular,and whole stimulated saliva in human [21]. According to Folch et al., in this method, the tissue is homogenized with Chloroform/methanol (2/1) to a final volume 20 times the volume of the tissue sample and after dispersion the whole mixture is agitated during 15–20 minutes in a orbital shaker at room temperature. The homogenate is either filtrated or centrifuged to recover the liquid phase. The solvent is washed with 0.2 volumes (4 mL for 20 mL) of water or better 0.9% NaCl solution. After vortexing some seconds, the mixture is centrifuged at low speed to separate the two phases. After centrifugation and siphoning of the upper phase, the lower chloroform phase containing lipids is evaporated under vacuum in a rotary evaporator or under a nitrogen stream if the volume is under 2-3 mL [22]. Larsson's findings indicate that saliva lipids mainly consist of cholesterol esters, cholesterol, triglycerides, diglycerides, monoglycerides, and free fatty acids [21]. According to Hindy et al., the saliva gland parenchyma is considered to be a place of sterols biosynthesis, similar to those of liver, as it was proven by using thin-layer and gas-liquid chromatography [23].

Man et al. demonstrated changes of the pancreas morphology by hypertriglyceridemia. It seems that triglycerides (TGs) are stored in pancreatic islets, which inhibited glucose-induced insulin secretion, in part, via reduced glucokinase activity in the islets [24]. Several studies have suggested that pancreas and parotid gland may present, in part, morphological and functional similarities [25–28]. Therefore, Anderson et al. studied salivary tissues of diabetic male Wistar rats in order to distinguish the differences in intracellular lipids between diabetic and controls glands. Lipid accumulation within parenchymal cells depended on the type of the gland and their content of serous cells. Parotid and sublingual glands possess the greatest amount of lipids compared to submandibular gland. Their findings may explain the mild alteration of the submandibular gland in patients with hyperlipidemia contrary to the parotid gland's involvement [29].

Moreover, this report reveals that the hyperlipidemic diet may result to no statistically significant decrease of parotid weight, observed in the hyperlipidemic-experimental group compared to controls. It is of great interest that hyperlipidemia, except for lipid deposition and lymphocytic infiltration of parotid gland, causes alterations of the parenchyma as well.

Furthermore, simvastatin treatment resulted in a statistically significant decrease of serum lipid profile but to a statistically insignificant decrease of parotid gland weight. Statins seem to repair parotid gland's changes caused by hyperlipidemia, resulting eventually to gland remodelling [11].

Due to our findings, a close relationship between high plasma lipid levels and parotid gland enlargement may be concluded. This enlargement might be an indication of parotid gland's microstructural changes, and it may explain itxs observed, by other investigators, functional impairment. It is obvious that statins, reducing the serum lipid profile, may play a protective role in organs' injuries caused by hyperlipidemia by repairing the occurred alterations.

## Figures and Tables

**Table 1:** Mean and standard deviation of mean of total cholosterol (TC), triglycerides (TG), high density lipoproteins (HDL), low -density lipoproteins (LDL), parotid gland-liver-thymos-spleen-adrenal weight, and atheromatic index in the four groups (A1, A2, B1, B2).

Groups	A1	A2	B1	B2
TC mg/dL	54.71 ± 0.000*	65.25 ± 0.000*	391.14 ± 0.000*	346.25 ± 0.000*
HDL mg/dL	17.09 ± 0.000*	20.12 ± 0.000*	40.86 ± 0.000*	36.37 ± 0.000*
LDL mg/dL	25.29 ± 0.000*	27.25 ± 0.000*	324.86 ± 0.000*	285.25 ± 0.000*
TG mg/dL	62.29 ± 0.000*	89.12 ± 0.000*	128.00 ± 0.000*	123.00 ± 0.000*
Parotid weight mg	37.80 ± 2.208***	39.57 ± 6.607***	43.71 ± 2.556***	35.84 ± 3.568***
Atheromatic index	3.20 ± 0.98***	3.24 ± 1.01***	9.57 ± 0.34***	9.52 ± 0.33***

*P < .001, **P < .01, ***P > .01.

TC: A1/A2***, A1/B1*, A2/ B1*, A2/B2*, B1/B2*, A1/B2*.

HDL: A1/A2***, A1/B1*, A2/ B1*, A2/B2*, B1/B2*, A1/B2*.

LDL: A1/A2***, A1/B1*, A2/ B1*, A2/B2*, B1/B2*, A1/B2*.

TG: A1/A2***, A1/B1*, A2/ B1*, A2/B2*, B1/B2*, A1/B2*.

Parotid weight: A1/A2***, A1/B1***, A2/ B1***, A2/B2***, B1/B2***, A1/B2***.

Atheromatic index: A1/A2***, A1/B1***, A2/ B1***, A2/B2^∗∗ ∗^, B1/B2***, A1/B2***.

**Table 2:** Item Measurement parameters (statistical average value) for cells and ducts in the four groups of animals.

	Density (ave)	Density (std)	Diameter	Ratios (PER area %)	Aspect ratio
Cells					

Group A1	74.621	7.248	5.589	7.154%	1.481
Group A2	71.890	6.969	5.663	9.409%	1.472
Group B1	76.413	7.722	6.658	5.660%	1.531
Group B2	78.066	7.950	5.877	12.361%	1.546

Ducts					

Group A1	130.849	35.091	33.736	1.545%	1.281
Group A2	131.985	37.292	34.437	1.481%	1.301
Group B1	135.055	36.146	41.140	0.633%	1.218
Group B2	135.064	36.740	40.838	2.388%	1.369
